# Coping with the invisible impacts of COVID-19 in a context of polycrises: wellbeing strategies of marginalised urban and Indigenous Brazilian youth

**DOI:** 10.3389/fpsyg.2025.1512893

**Published:** 2025-08-12

**Authors:** Susanne Börner, Leandro Giatti, Luciana Bizzotto, Michele Rocha El-Kadri, Ana Elizabeth Sousa Reis, Júlio Cesar Schweickardt, Peter Kraftl, Lauren Andres

**Affiliations:** ^1^University of Birmingham, Birmingham, United Kingdom; ^2^University of São Paulo, São Paulo, Brazil; ^3^Fiocruz Amazônia, Manaus, Brazil; ^4^University of Brasilia, Brasilia, Brazil; ^5^University College London, London, United Kingdom

**Keywords:** youth wellbeing, *bem viver*, COVID-19, decolonial—intersectional lens, Indigenous and urban youth, wellbeing strategies, polycrisis

## Abstract

The wellbeing of young people in Brazil is significantly impacted by interconnected challenges such as local and global inequalities, violence, the climate emergency, a loss of ancestral identity, and the increasing precarity of education and employment. These overlapping crises influence how young people make sense of their everyday lives, envision their futures, and adapt to wellbeing challenges. Public policies continue to inadequately address immediate and long-term wellbeing needs and local realities of youth in situations of vulnerability. Combining data from two research projects in São Paulo and the Brazilian Amazon, we explore the lived experiences and adaptive wellbeing strategies of marginalised Brazilian youth across urban and Indigenous communities during and after COVID-19 pandemic, based on youth-led survey data and participatory research. The article makes an important contribution to the field by proposing a decolonial counter-narrative to dominant Western understandings of youth wellbeing. Guided by the confluence of diverse worldviews from the margins, specifically Indigenous and urban Brazilian youth from the periphery, the article advances understandings of youth-led mental health from the perspective of the relational concept and practice of “*bem viver*” (“good life”). Results indicate that although youth mental health challenges, including anxiety and depression, were exacerbated during and after the COVID-19 pandemic. Youth however developed individual and collective youth-led self-care strategies. These were grounded in local realities recognizing solidarity, reciprocity, and interconnectedness as important pillars to maintain emotional stability and feel connected to others and the world around them. We recommend that interventions and policies to improve young people's inner states of wellbeing need to go hand in hand with community-oriented wellbeing strategies led by the principles of *bem viver* for collectively reimagining their futures.

## 1 Introduction

Young people worldwide, particularly in the rapidly transforming vulnerable territories and remote areas of the global South grow up into uncertain futures due to the overlapping and compounding polycrises of climate change, environmental degradation, violence, the cost-of-living, and the increasing precarity of education and employment (Andres et al., 2023; Börner, 2023; Kraftl, 2020; Kraftl et al., 2025). These interconnected and simultaneous crises amplify and accelerate the uncertainty of young people's futures (Henig and Knight, 2023) and have a significant impact on how young people make sense of their everyday lives and adapt to wellbeing concerns. As a result, anxiety and depression among young people in modern society have significantly increased (Henig and Knight, 2023; Samji et al., 2022; Germani et al., 2020). In countries of the Global South such as Brazil, young people living in contexts of (urban) vulnerability face systemic marginalization, adverse socio-economic and environmental conditions, and limited access to education, employment, and cultural or recreational spaces (Börner et al., 2020; Andres et al., 2023; Börner et al., 2023). These challenges are further intensified by intersecting global polycrises—such as climate change, economic instability, and public health emergencies—which disproportionately impact already marginalised populations. This creates a sense of unboundedness (Henig and Knight, 2023), where various critical conditions are deeply intertwined with emotional factors. For vulnerable youth, these overlapping crises further constrain life opportunities and deepen inequalities, posing additional challenges to being and living well.

Although youth mental health challenges were on the rise before the COVID-19 pandemic, the pandemic has created additional stressors with significant short-term and long-term mental health impacts on children and youth (Doom et al., 2023; Montero-Marin et al., 2023). Studies have found that although young people were physiologically less likely to be affected by the virus itself, the mental and social challenges created by the uncertainties, risks and anxieties of the pandemic led to a rise in depression, anxiety and emotional difficulties (Burton and Harwood, 2023; Camerini et al., 2022; Montero-Marin et al., 2023). Recent studies have also pointed to the need for further research to solidify and expand the evidence of mental health impacts of the COVID-19 pandemic on young people (Campion et al., 2020; Camerini et al., 2022). However, there is evidence that not all youth experienced poor mental health and some managed to cope better with the stressors of the COVID-19 crisis than expected (Doom et al., 2023), whether through individual or collective cultural practices, or through forms of “obsession” or “transgression” that are often dismissed as negative behaviors (Kraftl et al., 2025). To date, little research has been conducted on the factors associated with youth resilience and related variables such as self-efficacy, self-esteem, and life satisfaction during and after the COVID-19 pandemic (Doom et al., 2023).

In 2020, the UN declared mental health of underage groups a major public health priority, warning that mental health should be at the focus of post-COVID recovery measures [United Nations (UN), 2020]. However, mental health was not included as a priority in Brazil's National Contingency Plan for Human Infection by the COVID-19 (Noal et al., 2020). Moreover, studies on the lingering impacts of the pandemic on Brazilian youth in situations of marginalization, including urban youth in situations of vulnerability and Indigenous youth from the Amazon region, are rare. The voices and lived experiences of these young people that have been hit hardest by multifaceted crises including COVID-19 have been largely absent from research and policy (Silva et al., 2022; Kuhlman et al., 2021; Olivar et al., 2022). Even in diverse countries such as Brazil, most studies have focused on the (mental) health impacts and experiences of non-Indigenous youth (Zuccolo et al., 2023; Gimenes-Llort et al., 2022) and urban and Indigenous Brazilian youth have been excluded. This paper aims to address this gap by exploring not only on the mental health and wellbeing challenges caused by compounding crises, but also the self-care and community wellbeing strategies developed by vulnerable urban and Indigenous youth. Specifically, using the lens of the concept and practice of *bem viver* (“good living”), we explore how concepts and practices for relational living and interconnectedness can shape alternatives for wellbeing and well-living.

Over the past two decades, the prevalence of young people's mental health disorders has increased significantly, with most mental health disorders emerging before the age of 25. Empirical evidence indicates that this rise cannot be attributed solely to improved awareness or diagnostic practices but rather signifies a substantive and escalating public health crisis (McGorry et al., 2025; Lehmann et al., 2022). In the face of a looming global structural youth “mental health pandemic”, we argue that this requires new ways of conceptualizing being and living well from a youth-led perspective grounded in local realities and the confluence of diverse worldviews (Bispo dos Santos, 2023). According to Lemos et al. (2021), young people have the right to know and value different ways of acting, thinking, seeing the world and learning to relate to others. By considering the diverse lived experiences of both urban and Indigenous youth and understanding *what matters to these youth* in situations of marginalization, we aim to challenge everyday understandings and institutionalized (colonial) conceptualizations (Núñez, 2019) of youth “mental health” to explore more nuanced and culturally diverse dimensions of wellbeing. By bringing together diverse youth-led approaches for improving wellbeing in times of crises, this paper attempts to create a space for reflection and co-creation of wellbeing strategies from the margins.

In the following theory section, we discuss youth mental health challenges during and after COVID-19 in more detail before introducing the Indigenous concept and practice of “*bem viver*” (“good life”) as a decolonial counter-narrative (Kantner and Peixoto, 2023). In the methods section, we outline how we bring together datasets from two research projects to explore the lived experiences and adaptive wellbeing strategies of Brazilian youth in diverse marginalised communities: (1) PANEX- Youth, a participatory research project with young people from two of São Paulo's largest favelas, and (2) survey data gathered with diverse Indigenous communities in the Brazilian Amazon during the COVID-19 pandemic. In the results and discussion section, we (re-)conceptualize the lived experiences and adaptive wellbeing strategies of systematically excluded urban and Indigenous Brazilian youth through the lens of belonging, solidarity, collectivism, and reciprocity. We conclude by making recommendations for wellbeing interventions and policies, grounded in young people's everyday cultural and social realities and diverse knowledges.

## 2 Theoretical framework

### 2.1 Challenges and mental health impacts of COVID-19: diverse Brazilian youth experiences

#### 2.1.1 Youth mental health in contexts of vulnerability in Brazil

It is generally understood that a focus on childhood and adolescence is crucial when exploring mental health as many mental disorders start early in life and adversely impact lives into adulthood (Camerini et al., 2022). Brazilian youth in situations of vulnerability have historically been stigmatized and deprived of access to quality education, causing a lack of employment perspectives, a heightened anxiety about the future, as well as low self-esteem and sense of self-efficacy (Börner et al., 2020; Zuccolo et al., 2023). Both Indigenous and non-Indigenous Brazilian youth also express anxiety about the destruction of nature due to unplanned urban growth, climate change impacts, and an increased disconnection from ancestral knowledge (Börner, 2023; El Kadri et al., 2021; Verçosa et al., 2023).

Studies estimate that in Brazil, suicide, often linked to drug and alcohol abuse, is the fourth leading cause of mortality among young people between the ages of 15 and 29 (Razzouk et al., 2020). Community mental health services are unequally distributed across the country, and it is estimated that only 20% of children and youth with mental health needs in wealthy areas of Brazil's cities have access to mental health support (Zuccolo et al., 2023; Razzouk et al., 2020). This particularly limits the access to mental health services for young people in situations of deprivation to emotional regulation skills, cognitive reappraisal, problem-focused coping (Doom et al., 2023). The COVID-19 pandemic has further exacerbated young people's everyday struggle for survival, food, shelter, and physical safety over possibilities of self-expression (Courtney et al., 2020; Furtado, 2021).

#### 2.1.2 Youth in crisis: disrupted routines and relationships during COVID-19

During COVID-19, young people were particularly affected by the disruption of social activities and interactions, including social distancing measures and school closures that interrupted interaction with peers and the navigation of relationships outside the family home (Tebet et al., 2021; Camerini et al., 2022; Doom et al., 2023; Andres et al., 2025). At the same time, they experienced the postponement and cancelation of important life events (Kuhlman et al., 2021; Kraftl et al., 2025) and witnessed other stressors such as financial, and sickness-related challenges, including essential financial challenges due to a loss of family income, food insecurity, or the COVID-19 related death of primary caregivers or close family members (Doom et al., 2023; Jaeger et al., 2021). In Indigenous communities, the loss of Indigenous elders to COVID-19 resulted in a significant loss of oral knowledge and customs as well as a lack of guidance for young people (Mendes et al., 2022).

In Brazilian cities, school closures were particularly challenging. With closures lasting over 40 weeks, Brazilian youth were among the most affected globally by the impact of COVID-19 lockdowns, with an estimated 44.3 million students deprived of nearly all in-person classroom instruction during this period (Zuccolo et al., 2023; Silva et al., 2022). In addition, the education of young people was severely affected by digital inequalities from the early days of the pandemic, further deepening existing disparities (Börner et al., 2020). School closures also eliminated critical environments of protection and support for young people facing difficult social and family circumstances, exacerbating emotional and social challenges during periods of confinement. Growing up in conflict-ridden households during this time significantly increased the risk of adverse mental health outcomes (Courtney et al., 2020; Samji et al., 2022).

#### 2.1.3 COVID-19, urban marginality, and the shift to digital socialisation

Disruptions to daily routines increased the risk of mental health disorders and depressive symptoms, particularly across vulnerable populations (Zuccolo et al., 2023). A review of literature on young people's everyday experiences and mental health impacts during the COVID-19 pandemic shows that at the level of the individual, children and youth struggled with the impacts of a loss of “activities that provide structure, meaning, and a daily rhythm” (Courtney et al., 2020, p. 688; Samji et al., 2022; Doom et al., 2023). Changes in habits during the pandemic also led to more sedentary lifestyles and excessive screen time (Camerini et al., 2022; Schmidt et al., 2020). Despite digital inequalities (Andres et al., 2025; Börner et al., 2023), young people in Brazil increasingly turned to online spaces for leisure and social activities. This growing integration of digital media blurred the boundaries between their online and offline interactions, shifting peer socialization and recreation predominantly to virtual environments (Bork-Hüffer et al., 2021).

This shift was intensified by the limited availability of leisure options in Brazilian urban peripheries, where access to public spaces and natural environments is often severely restricted (Börner et al., 2020). In areas of urban sprawl, slums and other informal settlements in Brazil are typically characterized by high population density, precarious infrastructure, combined with a lack of green areas and public amenities (Hadfield-Hill and Zara, 2019; Sahni and Kumar, 2021). While research highlights the positive psychological benefits of nature connection during the pandemic (Haasova et al., 2020; Sahni and Kumar, 2021), young people living in these rapidly transforming urban margins—such as those in São Paulo—face significant barriers to engaging with greenspace (Campello Torres et al., 2021; Jaeger et al., 2021). Nature is these settlements is frequently seen as “hampering” development, leading to destructive human-nature relationships, and diminishing existing opportunities for everyday encounters and interactions. During the COVID-19 pandemic, the challenge of accessing nature spaces in Brazilian cities was further heightened by the closure of—the already scarce—public greenspaces during the periods classified as periods of major transmission of the virus. Hence, young people had to find alternatives to nature-based strategies for stress relief and relaxation based on human-nature interactions and nature connectedness (Tiriba and Profice, 2019; Yeung and Yu, 2022; Robinson et al., 2020; Sahni and Kumar, 2021). For many young people, the shift to the digital therefore also became an escape from the social and physical stressors of urbanization and a key source of peer support (Gruebner et al., 2017; Doom et al., 2023; Kraftl et al., 2025).

#### 2.1.4 Intersecting crises: wellbeing, territory, and identity among Indigenous youth

Indigenous youth in the Brazilian Amazon—both in urban areas and traditional communities—were particularly vulnerable during the pandemic, facing elevated health risks, cultural erosion, deteriorating mental health, and increased territorial violence, all amid limited institutional support (El Kadri et al., 2021; Ro, 2023). The overall precarious wellbeing situation was aggravated by a limitation of access to health services. Under the argument “isolating to protect”, the federal government suspended the entry of health services into Indigenous territories. As many Indigenous areas are only accessible by river, access to specialized healthcare for these communities was seriously impacted (Mendes et al., 2022). Social isolation measures also aggravated pre-existing social vulnerabilities due to a lack of income, sanitation, and medical services, adding another layer to pre-existing mental health stressors (Ro, 2023). This also raises heightened concerns about the mental and emotional wellbeing of Indigenous youth as the pandemic restrictions implied reduced support for young people struggling with alcohol and drug abuse and domestic violence (El Kadri et al., 2021).

At the same time, illegal mining invasions and deforestation surged during the pandemic as the government administration under Bolsonaro scaled back on environmental enforcement in Brazil leading to the highest rates of forest fires in over two decades (Vale et al., 2021; Mendes et al., 2022). The Bolsonaro Federal government's pandemic-era policies of socio-environmental neglect and exploitation in Brazil (Mendes et al., 2022; Vale et al., 2021) have intensified longstanding conflicts rooted in oppressive and exclusionary structures, largely due to their failure to acknowledge and engage with Indigenous worldviews and aspirations. Conversely, legitimate responses from diverse local initiatives have shown that collaborative efforts and cosmopolitical approaches (considering the interconnectedness of all people, species, and non-human entities) can effectively address the complex and multifaceted challenges posed by emerging diseases such as COVID-19 (Olivar et al., 2022).

Indigenous youth also face additional pressures arising from the complex dynamics of ongoing migration to urban centers in the Amazon region, often driven by the pursuit of education, healthcare, and employment opportunities (Coimbra Jr and Santos, 2000). Interactions between different ethnic groups, as conceptualized by Barth (1969) as *interethnic borders*, can generate additional tensions and challenges for Indigenous youth in cities, weakening traditional practices and generating fluid identities that can be deeply antagonistic and lead to mental suffering. The COVID-19 pandemic further exposed and intensified these challenges, as weakened ties to home communities heightened their social and emotional vulnerability in urban spaces. For those relying on traditional livelihoods such as handicraft production, the economic restrictions imposed during lockdowns limited income-generating opportunities, increasing financial strains during lockdowns (UNICEF Brazil, 2025).

**Figure 1:**
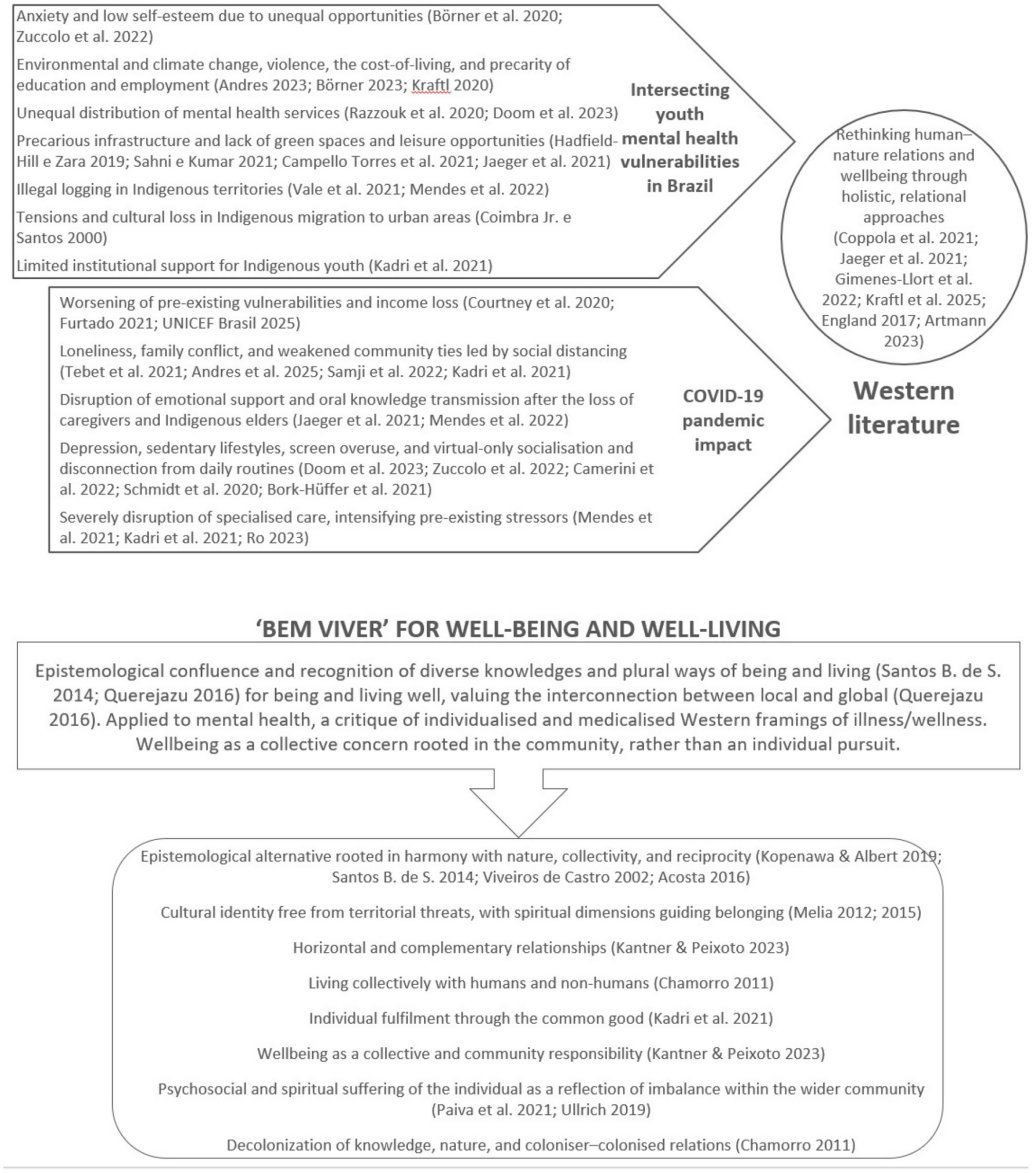
Overview of the theoretical framework.

Despite these multiple and interconnected challenges, Indigenous youth have found ways to redefine their presence in urban areas, using social mechanisms to adapt their use of available spaces toward practices of being and living well (Verçosa et al., 2023; Alves et al., 2023). We introduce relational Indigenous cosmovisions of being and living well in Section 2.2. Although the intersection of ancestral worlds and knowledges (and their loss) with the contemporary urban environment creates often conflicting, encounters (El Kadri et al., 2021; Verçosa et al., 2023), Indigenous youth also play a key role in preserving their communities' ancestral heritage and traditional knowledge, while navigating the challenges of modernity. An overview of the theoretical framework is illustrated in Figure 1.

### 2.2 “*Bem viver*”: toward diverse relational cosmovisions of being and living well

#### 2.2.1 Critiques of western mental health models

While the COVID-19 pandemic made local and global inequalities and social injustices explicit, it has also reinforced the need for community and traditional strategies to deal with health risks (El Kadri et al., 2021). To date, most studies have explored the “mental health” impacts of the pandemic from a (Westernized) understanding of mental health as a “state of wellbeing in which an individual realizes his or her own abilities, can cope with the normal stresses of life, can work productively and is able to make a contribution to his or her community” (Camerini et al., 2022:1). From a Western perspective, conceptualizations of mental health are generally based on the understanding that it is possible to separate the health of the body, from the emotional health, the spiritual health, and the health of the environment Moreover, Western medicine tends to individualize and medicalize mental illness, framing it as the psychosocial and spiritual suffering of the individual rather than as a reflection of imbalance within the wider community (Wamsler et al., 2021; Paiva et al., 2021; Ullrich, 2019).

We argue that decolonizing conceptualizations of “mental” health requires challenging dominant notions of psycho-spiritual wellbeing that render diverse (marginalised) knowledge(s) from the global South invisible or impose Western understandings over different ways of knowing (Núñez, 2019; de Sousa Santos, 2014). Young people from Indigenous communities in the Brazilian Amazon (and elsewhere) suffer immensely from the dismantling of traditional knowledge as they stop practicing traditional approaches to healthcare and wellbeing, replacing them with dominant Western “cosmology”, religious beliefs, and Western health care theories (El Kadri et al., 2021). This break with Indigenous culture and the attempt to deny their traditions, conceptualizations and beliefs directly affects the psycho-spiritual balance of young people, causing emotional destabilization and an identity crisis (El Kadri et al., 2021).

From an Indigenous perspective, there is no reason to name or separate different types of health or wellbeing: individual wellbeing is considered interrelated with that of the environment, whether it be elements of nature, community or familiar life, or the spiritual (El Kadri et al., 2021; Ullrich et al., 2022). Principles and practices of Indigenous interconnectedness center values such as community connectedness and a sense of belonging within the more-than-human collective (Ullrich, 2019; Ullrich et al., 2022). We therefore challenge the terminology centered on Western conceptualizations of mental health which have dominated scientific discourse in mental health to address the mental health crisis of the modern world at the expense of other, diverse conceptualizations of wellbeing.

We argue that there is an increasing need to transcend conceptualizations of wellbeing that perceive (and reproduce) a split on a structural level between external and internal (emotional) worlds or wellbeing dimensions (Artmann, 2023), and that are centered on the (human) individual at the expense of more relational understandings. In the following, we therefore introduce the Indigenous concept and practice of “*bem viver*” (“good life”) as an alternative cosmology and decolonial counter-narrative (Kantner and Peixoto, 2023) to contextualize and (re-)conceptualize the lived experiences and adaptive wellbeing strategies of both marginalised urban and Indigenous Brazilian youth. Our understanding of wellbeing draws on Indigenous cosmopolitics (De La Cadena, 2019), which stresses the interconnectedness of humans, nature, and communal wellbeing, challenging the ontological divide of modern Western thought (Ullrich et al., 2022; Kopenawa and Albert, 2019).

#### 2.2.2 Indigenous perspectives on being and living well

Centring the work of a variety of (Indigenous) scholars on the concept of “bem viver”/”buen vivir” and good living (see Kopenawa and Albert, 2019; de Sousa Santos, 2014; Viveiros de Castro, 2002; Acosta, 2016), we intend to use the concept and practice of “bem viver” as rooted in Latin American Indigenous philosophies, epistemologies, and communities' perspectives on harmony, sustainability, and community. According to Melia (2012, 2015), the concept of *bem viver* describes a way of being or living well as a state of joy and satisfaction, peace, and harmony with nature and with the community, based on reciprocity and respect. It also includes an understanding of a cultural identity that is fully experienced, free from threats in the territory, including spiritual aspects that guide belonging and reciprocity in a shared life experience. *Bem viver* also represents the quest to understand that building a different world requires recognizing that relationships must be horizontal and based on reciprocity and complementarity (Kantner and Peixoto, 2023), a premise that is lacking in the foundations of capitalism and colonialism (Verçosa et al., 2023). Essentially, *bem viver* is about living well together, in community with all beings (human and non-humans) (Chamorro, 2011; Kantner and Peixoto, 2023; Monteiro and McCallum, 2013), finding individual contentment in considering the collective (El Kadri et al., 2021). Kantner and Peixoto (2023) also argue in favor of treating *bem viver* as an “ongoing process of struggle” (p.473) rather than “something ancestrally frozen in the past” (ibid.), recognizing its multiple meanings and contextualization's.

#### 2.2.3 Muted modernities and the relational (re-)connection

The *bem viver* concept is also an attempt to rethink human-nature relations and wellbeing holistically and relationally. Rosa (2019) describes modern Western (urban) societies as “muted”, shaped by rational and instrumental relations and a loss of sensitivity to spiritual consciousness (Rosa, 2019; Artmann, 2023). Indigenous critiques (Ullrich, 2019) argue that spirit and spirituality are frequently reduced to Western categories like “culture” or “religion”, overlooking that, within the interconnected web of life, our existence as spiritual beings is a core aspect of what it means to be human (ibid.). However, the notion of “spirituality” has increasingly been employed across Western contexts to describe a unifying a state of one-ness connecting the mind, body, and the spirit as well as the individual, society, and nature (Coppola et al., 2021; Jaeger et al., 2021). Some of the Western literature on positive adaptations to the COVID-19 crisis has also pointed to the importance of spiritual coping (in terms of believing in and praying to a higher power) as a protective factor to find meaning and purpose in times of grief, leading to a better self-esteem and stronger social ties as well as socio-emotional resilience (Gimenes-Llort et al., 2022; Coppola et al., 2021; Kraftl et al., 2025).

From a Western perspective, relational wellbeing approaches have been framed within the perspectives of transpersonal spirituality and eco-psychology (Artmann, 2023; Müller et al., 2023). Ecopsychology for instances emphasizes the importance of transpersonal relationality and highlights the necessity for modern society to cultivate emotional connections that foster a deeper bond with nature, recognizing the importance of the interconnectedness of all living beings and recognizing the need to transcend human centeredness (England, 2017; Artmann, 2023). In response to a perceived spiritual “emergence/emergency” (England, 2017) in modern (urban) society, these approaches aim to heal the disconnection from self, the purpose of life, and nature, by reintegrating physical, emotional, social and spiritual dimensions of human-nature reconnection. In Indigenous relationality and interconnectedness frameworks such as *bem viver*, these dimensions have however never been separated in the first place. The concept and practice of *bem viver* emphasize that wellbeing is a collective concern rooted in the community, rather than an individual pursuit (Kantner and Peixoto, 2023; El Kadri et al., 2021).

When approaching *bem viver* and mental health in the context of marginalised Indigenous and non-Indigenous youth living in urban environments or communities, it is also imperative to transcend the mere analysis of their urban reality, as the challenges faced by these groups are intrinsic to broader processes. The urban life of these young people encompasses a multiplicity of dimensions, ranging from the urban space to ethnic and identity boundaries, including ancestral expectations (especially for Indigenous youth) and the dynamics of power and domination (Verçosa et al., 2023). Indigenous leaders such as Krenak (2019) challenge us to look at nature without the lens of capitalism, alerting us to the fact as a product of colonization, the city has been seen as opposed to nature which has been associated with threats and dangers. He therefore proposes a reconnection with the vast living world that encompasses all forms of life (Krenak, 2019; Verçosa et al., 2023).

#### 2.2.4 Epistemological pluralism: toward a confluence of knowledges

*Bem viver* implies a respect for other worldviews, other universes of meaning, based on a decolonization of knowledge, of nature and of colonized and colonizers (Chamorro, 2011). The concept of *bem viver* has however been criticized as potentially creating new hierarchies of knowledge. Some scholars (see Cochrane, 2014; de Zaldívar, 2017) have raised concerns that *bem viver*, while intended as a decolonial and pluralistic alternative to Western development models, could inadvertently create new hierarchies. For instance, critiques argue that by seeking to reject Western rationality, there is a risk idealizing and essentializing Indigenous cosmovisions. However, our intention is not to advocate for new ontological hegemony. Through the confluence of marginalised knowledges, we aim to eradicate any existing (Western dominated) knowledge hierarchies to contribute to more epistemological diversity as a recognition of diverse lived experiences and cosmovisions (de Sousa Santos, 2014; Querejazu Escobari, 2016). In this sense, the fundamental starting point is the cultivation of intellectual autonomy, enabling the ability to think for oneself and dream of one's own ideals to re-signify the past in the present.

We consider decolonization an ongoing and gradual process, as colonization is deeply rooted in social structures and thinking. Dismantling the political, economic, social, cultural and mental systems of exclusion and power that are still entrenched in patriarchal and colonial histories is therefore an integral part of the process (Verçosa et al., 2023; Askins, 2018). Moving toward more nuanced understandings of psycho-spiritual wellbeing based on diverse lived experiences and worldviews is an important step toward respecting other possibilities of (well-)being and relating to self, others, and nature—both physically, emotionally, and spiritually. Only then we can fully open our minds to appreciate the interconnections of the local and the global (Querejazu Escobari, 2016).

In the following section, we will now present the two case studies from São Paulo and the Brazilian Amazon to explore the confluence of young people's perceptions of wellbeing and their adaptive actions during COVID-19 from the perspective of urban and Indigenous youth.

## 3 Methods

This article explores the wellbeing experiences of marginalised youth during the COVID-19 pandemic based on two case studies: (1) the PANEX-Youth project based on participatory research with 28 young people in São Paulo's urban periphery, and (2) Fiocruz survey data gathered with 533 Indigenous communities in the Brazilian Amazon region. Both case studies bring in diverse, complementary perspectives and methodologies for joint learning to develop new conceptual and policy frameworks for youth wellbeing. They also highlight the diverse, yet similar, challenges and adaptations that young people faced during the COVID-19 pandemic and post-pandemic. Both cases were chosen based on previous international collaborations involving Brazil and UK research teams, bringing together researchers with a focus on young people, wellbeing and polycrises research.

### 3.1 PANEX-youth

**Figure 2:**
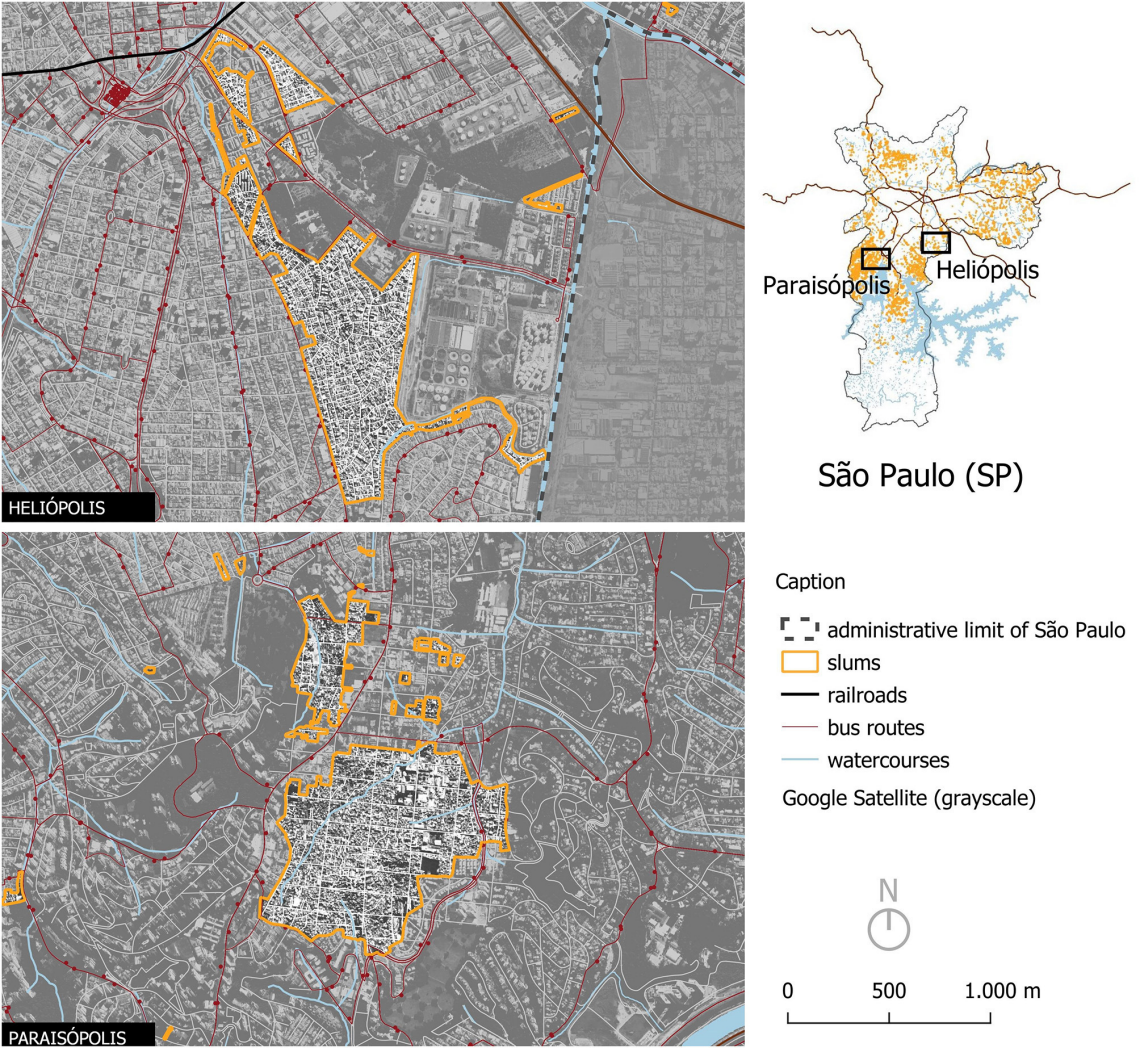
Map of the communities of Paraisópolis and Heliópolis in the city of São Paulo (SP, Brazil). *Source* Author's elaboration with data from the São Paulo City Hall (2023).

As part of the PANEX-Youth project (2022–2024; see Andres et al., 2025; Kraftl et al., 2025), an international collaboration across the UK, Brazil, and South Africa, funded by the Transatlantic Platform and the São Paulo Research Foundation, participatory research was conducted with 28 marginalised young people aged 14–23 years in two of the largest favelas (Paraisópolis and Heliópolis) in the urban periphery of São Paulo to explore young people's lived experiences with COVID-19 (Figure 2). Data were collected after the peak of the pandemic, between 2022 and 2024.

The 28 young people were engaged through two community-based organizations: Pro Saber in Paraisópolis and Observatório De Olho Na Quebrada in Heliópolis. These partners were identified through an earlier mapping stage, which involved interviews with local groups supporting children, youth, and families during the COVID-19 pandemic, using a snowball sampling approach. Both organizations had longstanding experience working with young people and were invited to collaborate in the participatory phase of the study. Therefore, the selected youth were already involved in regular activities within these spaces.

To support the research process, a “dissemination course” was delivered to both groups, consisting of five 3-h sessions held between June and August 2023 at each location. Participants received certificates issued by the University of São Paulo (USP). Activities followed a World Café format (Fouché and Light, 2011), guided by questions developed by the international team and complemented by a playful QandA game to promote collective reflections between the young participants. In addition, a *visual web* tool (Kraftl et al., 2019) was used. The visual web exercise used visual representations, such as images, and their interconnections to explore individual and subjective visual expressions of young people's lived experiences. The visual web exercise was applied during the final session of the dissemination course. Participants created individual compositions using selected images and drawings, which they later presented to the group Using a mix of drawing, photo-annotations, mapping, and interactive feedback, the visual web tool enabled participants to capture and share their observations and experiences with their local environment. The activity was conducted in a collective setting, with researchers facilitating small-group and one-on-one conversations to deepen participants' reflections.

Group discussions, supplemented by field notes, were audio-recorded and transcribed. All data from the visual web, world cafes, and interviews was coded in NVivo using thematic analysis. Data was analyzed around the PANEX-Youth core themes (food, leisure/play, and education), everyday wellbeing concerns and adaptative wellbeing strategies during the COVID-19 pandemic. The process began with thematic, comparative coding involving the larger international team, followed by open coding to capture context-specific themes emerging from the Brazilian fieldwork. All files were anonymized and archived in the University of São Paulo's digital repository. Ethics approval for the PANEX-Youth was granted for the project by the University of São Paulo's ethics committee.

### 3.2 Fiocruz survey in the Brazilian Amazon

**Figure 3:**
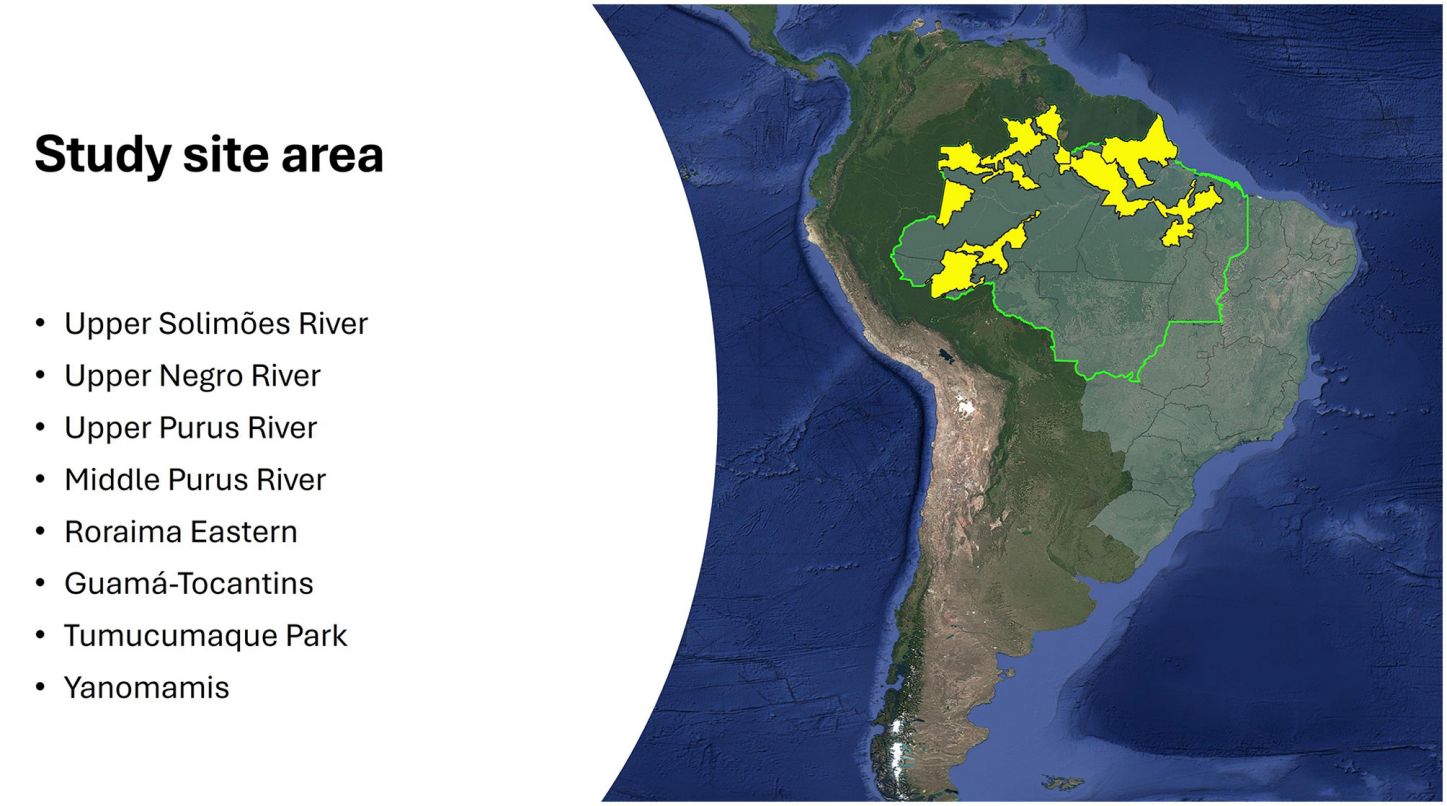
Research area covered by the survey [Fundação Oswaldo Cruz; Instituto Leônidas and Maria Deane (FIOCRUZ), 2021].

The Fiocruz survey on Knowledge, Attitudes, and Practices (KAP) was conducted within the project “Mental health of Indigenous populations in the Brazilian Amazon in the context of COVID-19”. The project was the result of a partnership between the United Nations Children's Fund—UNICEF/Brazil, the Laboratory of History, Public Policy, and Health in the Amazon (LAHPSA) of the Leônidas and Maria Deane Institute (ILMD)/Fiocruz Amazônia, and the Coordination of Indigenous Organizations of the Brazilian Amazon (COIAB). The study and the project received financial support from the United States Agency for International Development—USAID. The survey was carried out with 533 Indigenous youth aged 15–22 years from diverse Indigenous communities in eight target areas of the Brazilian Amazon Region (Figure 3).

The survey was designed by the Laboratory of History, Public Policy, and Health in the Amazon (LAHPSA). It was divided into three blocks including questions on sociodemographic data, knowledge about mental health and information about COVID-19 to identify the wellbeing concerns, attitudes, and practices among Indigenous youth during the peak of the COVID-19 pandemic. The aim of the survey was to develop a national training to address mental health to support the health care workers who work with Indigenous communities. Resonating with the participatory approach of the work in São Paulo, local young people were mainly responsible for conducting the survey, including meetings with community leaders, shamans, parents, and other young people themselves.

The main strategy used was to send a link to the questionnaire, made available via google forms, to young people's mobile phones. Owing to difficulties accessing the internet, an additional printout of the questionnaire was sent to the communities. Another challenge was language; to address this issue the questionnaire was applied by the community members themselves to ensure an accurate translation into the respective local languages. Data were analyzed using descriptive statistics and thematic analysis within the broader categories of behaviors, practices, and attitudes. These including health care practices, sociocultural attributes, contact with COVID-19 and coping measures. After an initial analysis of the data, a virtual meeting was held with the young people responsible for the survey to contextualize the findings and clarify certain terms and expressions. Overall, it is important to mention that Indigenous young people were primarily responsible for the wide reach of this survey. In addition to administering the survey, they enriched the empirical base of the study with narratives about how it was carried out, the impacts of the mobilization within the communities, images, and the contextualization of the data to ensure its reliability.

## 4 Results

### 4.1 Case study 1: the invisible youth mental health pandemic in the urban periphery of São Paulo

#### 4.1.1 Lack of green and leisure spaces

It is almost impossible to construct a shared understanding or meaning of the COVID-19 impact on young people even within the same city, as the lived realities of young people in situations of vulnerability were markedly different from those who had the privilege to self-isolate. This reinforces the importance of deconstructing the notion of “the youth” by echoing voices from a *diversity* of lived experiences. Young people's experiences in the urban periphery of São Paulo revealed several key factors that negatively affected mental health during the pandemic. These included social isolation, lack of access to green and leisure spaces, lack of emotional support, grief from the loss or severe illness of loved ones, and the pressure to grow up too fast.

Young people raised the observation that for many of them, life had to go on despite the pandemic, either due to economic pressure to make an income or as a coping strategy to avoid falling into depression. As a young girl from Heliopolis explains: “*I don't think there was a pandemic here, so I don't think people have much to say because for us here there was no time to isolate yourself. There was no way you could isolate yourself. There was no way you could stay at home and study. I think for everyone, either it was a peak moment when everyone took it upon themselves and started making an income on their own, took the initiative to do something because there was too much time on their hands, or the person sank into mental illness, you know, started having depression, anxiety, developed this and had a bad time.”* This also shows how structural shortcomings such as the lack of adequate conditions for young people to stay at home, due to overcrowded housing or a lack of quiet learning space, were part of young people's realities (Börner et al., 2020).

Already, pre-pandemic, young people from the urban periphery of São Paulo suffered from a lack of adequate options for leisure activities or spaces—beyond soccer pitches and child playgrounds—failing to attend to the plurality of their needs in terms of age, gender and cultural diversity (Roberto and Uvinha, 2021). This worsened during the COVID-19 pandemic due to the closure of open and public spaces or shopping centers where they could meet their friends. The lack of leisure spaces for peer interaction in urban peripheries contributed to youth mental health struggles, leading many to rely more heavily on social media as a coping mechanism. Young people equally reported struggling with the challenge of overcrowded living conditions, which negatively impacted their ability to study and made it impossible to self-isolate (Börner et al., 2020).

This lack of quality leisure spaces for young people during the pandemic reflects the lack of space for young people in society and politics (Andres et al., 2025). In conversations with the young participants, they described leisure during the pandemic as largely limited to screens—particularly video games and mobile games—or, for those who disregarded social distancing, the streets, including *baile funk* and local sports courts. At the same time, young people's experiences of (chronic) waiting and finding ways of passing time in an environment with limited opportunities highlighted the importance of waiting as a social experience and resistance (Jeffrey, 2010).

#### 4.1.2 Screens, streets, and self-care

In the absence of governmental interventions, young people also developed an array of individual and collective self-care and solidarity strategies perceived to alleviate depression and anxiety, by transforming their present realities online and offline (Paiva et al., 2021). Some of the young people from Paraisopolis reported using YouTube in a more “active” way for learning and teaching, for instance by creating a YouTube channel to teach others how to play certain games (Kraftl et al., 2025). This is shown by the following comments of participants: “*I played games to make it feel real and to make new friends, since I couldn't meet anyone in person. I made virtual friends to escape loneliness and avoid falling apart”* (Boy from Paraisópolis). However, young people self-identified the intensive use of screens and social networks as elements that played a central role in creating mental health challenges by reducing their self-esteem.

Due to the absence of green areas or open and public spaces for stress reduction and relaxation (Camerini et al., 2022), opportunities for mindfulness and physical exercise for young people in the urban peripheries were limited. At the same time, apart from excessive screen time, many of the young people developed what they called “*surtos*” (English: obsessions) to maintain emotional stability and release energy, e.g., by excessively engaging in activities such as making bracelets or practicing judo and capoeira. Here, for some of the young people, outdoor backyards or the street became hubs for sports and other activities for distraction. A boy from Paraisopolis commented: “*One of my best ways to enjoy myself was playing football, even during the pandemic. I kept playing, but I was afraid something bad would happen*.”

Young people living in peripheral areas already face increased stigmatization and precarity, as their presence in public streets is often viewed as a threat to public safety while their fundamental right to survive is at risk (Katz, 2018). Young people from the project expressed detrimental mental health impacts linked to the stigma of being a resident of a favela, living in poverty and with limited opportunities. They perceived that their lives mattered less than that of “wealthy” residents, making them second-class citizens from the authorities' perspectives. The COVID-19 pandemic intensified both the overall climate of uncertainty and the insecurity of public spaces, aggravating psychosocial suffering especially in disadvantaged communities (Börner et al., 2020; Punch, 2016; Paiva et al., 2021).

#### 4.1.3 Lack of social emotional support

Young people from the urban periphery particularly also mentioned the lack of social protection and emotional support as key for reported mental health breakdowns during the pandemic. A young woman from Heliopolis explained that because of the mental health crisis linked to the COVID-19 pandemic, “*I closed myself off from the world, I hate it to the point of breaking things*”. To help foster better socio-emotional coping strategies and behavioral regulation practices, there is a need for adequate youth mental health support for young people beyond individualizing and medicalizing psychosocial suffering (Wamsler et al., 2021; Paiva et al., 2021). COVID-19 exacerbated the underlying structural adversities of growing up in the urban periphery of São Paulo (Börner et al., 2020), highlighting the structural gap in access to adequate institutional psychological support (Zuccolo et al., 2023).

Yet, although the COVID-19 pandemic gave rise to “a perfect storm of psychosocial suffering” (Paiva et al., 2021, p. 1,464), in the absence of governmental and institutional responses, young people in situations of vulnerability developed a series of strategies to maintain emotional stability, pass time, and feel more connected to their community. It is however important to point out that young people should not be “expected” to simply be resilient without adequate support from caregivers and society (Doom et al., 2023). This was supported by young people's perceived experience of having to grow up too fast during pandemic times.

#### 4.1.4 Growing up too fast during the COVID-19 pandemic

Several young people felt forced to grow up too quickly, lacking the socialization, peer support, and sense of belonging crucial for transitioning into adulthood (Denworth, 2020). They also expressed the feeling of missing out on crucial life events and a sense of grief for the essential time lost for development and self-exploration during crucial teenage years. A young woman from Heliópolis reflected:

“*I went to only one university party, less than a week and a half before the lockdown isolation, after which part of my youth was stolen. I miss what will never come back. I feel like I've lost the opportunity to be young. I had to mature very quickly in a short space of time. [...] There are scars that will last forever. Today I understand the fear of loneliness.” (Girl from Heliópolis)*

This shows that the invisible mental impacts and long-term psychological effects of the COVID-19 pandemic extend far beyond the pandemic times (Adorjan and Stubbe, 2023).

At the same time, the COVID-19 reality for young people in São Paulo's favelas was marked by contradictions—although schools and other public places like parks were closed, they felt that in their immediate environment the COVID-19 pandemic did not exist. In deprived neighborhoods, basic needs for survival often took precedence over self-realization and the lack of income from other daily jobs and pushed people to develop different initiatives such as starting to make chocolates, selling clothes, or making an income as hairdressers to guarantee a contribution to the family income.

#### 4.1.5 Grief and bereavement

Young people also reported struggling with situations of individual and collective grief due to bereavement during the COVID-19 pandemic or fear of loss. One young girl from Heliopolis explained how her aunt and uncle both passed away because there were not enough hospital beds. She had to organize a coffin, write an obituary, and notify her family. Besides, her uncles' coffin was sealed, and she was deprived of properly saying goodbye to the body. With a lack of psychological and spiritual support during times of individual and collective grief, young people found it challenging to maintain a sense of meaning or actively engage in an active grieving process rather than avoiding their emotions (Coppola et al., 2021; Jaeger et al., 2021; Tschakert et al., 2023). This emotional suffering was captured by a girl from Heliópolis, who shared: “*My form of leisure was depression. I stayed in a foetal position, because during the pandemic I spent a lot of time alone*”.

Unsurprisingly, this feeling of loss was often associated with psychological distress, commonly described in pathologized terms (Paiva et al., 2021). In Heliópolis, many young participants understood the research process itself—which involved conversation circles focused on lived experiences—as an opportunity to process their hardships, reframe difficult moments, and recognize their own role in overcoming adversity. Recognition and belonging, fostered through active listening, emerged as key elements in supporting their mental health. This also extended to shared activities and collective work; many young people emphasized how simply being together and sharing daily life played an important role in promoting wellbeing.

#### 4.1.6 Adaptive wellbeing strategies: solidarity in adversity

**Figure 4:**
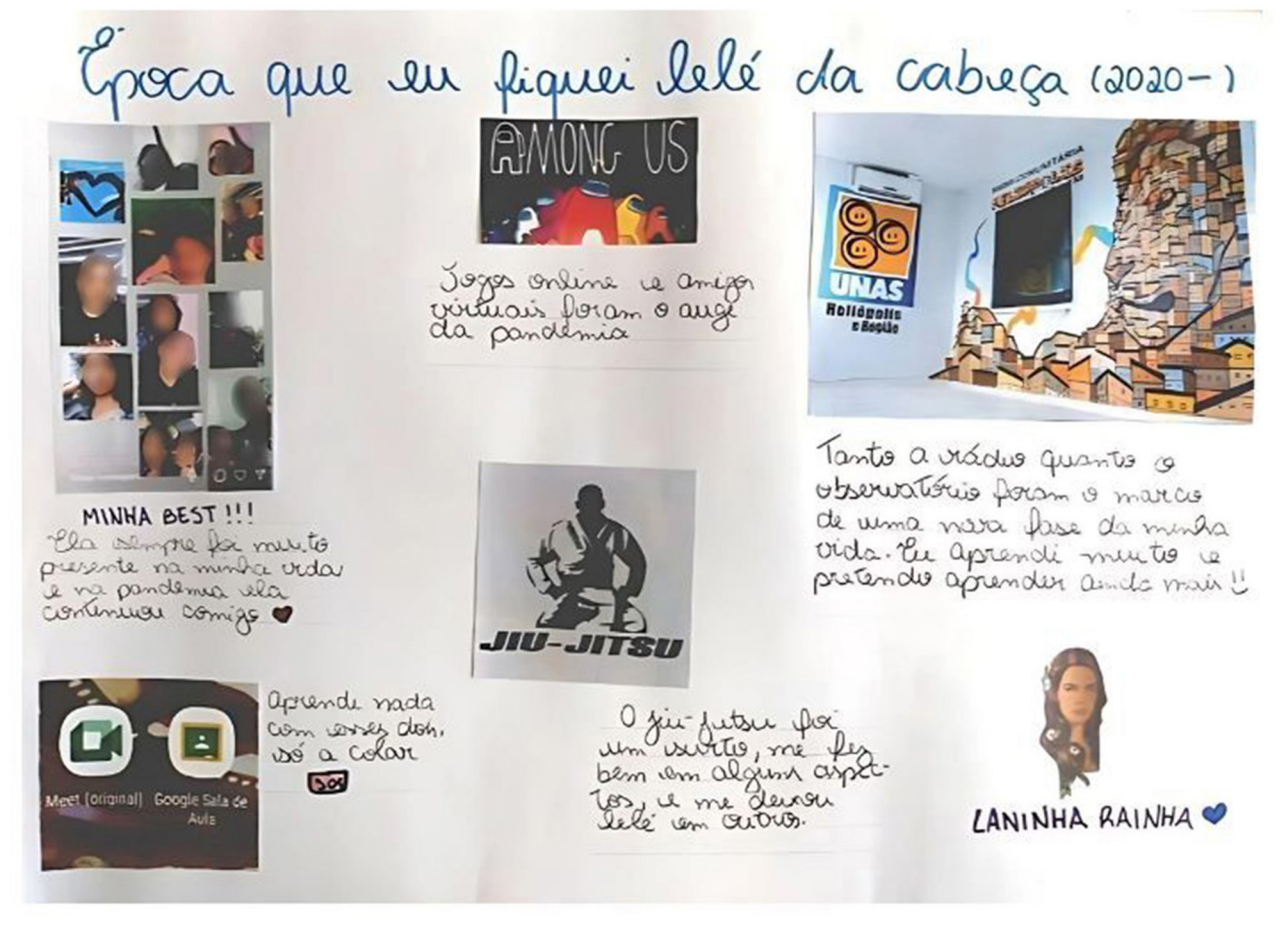
Example of a visual web on youth wellbeing practices developed by a girl from Heliopolis during the COVID-19 pandemic (PANEX Youth project). Caption (translation from originally written in Portuguese): Title “A time when I was out of my mind (2020)”; From left to right – “My bestie! She has always been very present in my life and during the pandemic she has stayed with me”; “Learning nothing but cheating from these two.”, “Online games and virtual friends were the height of the pandemic”; “Jiu-jitsu was an obsession, it was good for me in some ways and it made me crazy in others”; “Both the [community] radio station and the [De olho na Quebrada] Observatory marked a new phase in my life. I've learned a lot and I intend to learn even more”.

Young people pointed out that the larger structural processes that produce vulnerabilities, such as the inability to self-isolate, also led to the creation of solidarity networks within their communities. Such support networks, either peer-run or intergenerational, played a key role in alleviating individual suffering while helping young people to adopt other narratives of themselves, beyond limited beliefs in what life had prescribed for them. This also shows the importance of social ties and peer-support for an enhanced sense of self-esteem and self-efficacy (Doom et al., 2023). Figure 4 shows an example of a visual web production on wellbeing practices and solidarity networks developed by a girl from Heliopolis.

Such narratives showed that solidarity networks that emerged in the urban periphery during the pandemic were strongly experienced as networks of mutual affection, reciprocity, belonging, and emotional support (Furtado, 2021; Domingos et al., 2023). Young people who participated in the “*De Olho na Quebrada Observatory*” youth researcher collective in Heliopolis repeatedly stated how their participation in the group had helped them to maintain their mental health through the sense of collective support and connection. These informal and formal solidarity networks played a key role in giving young people the opportunity to draw on the “ordinary magic” (Masten, 2001) of their capabilities, relationships, and resources to protect and improve their mental health.

#### 4.1.7 Reinventing solidarity online

During the COVID-19 crisis, young people were however challenged to reinvent expressions of solidarity, increasingly using social media as a tool to stay in touch, network, and socialize via the means of WhatsApp, TikTok or online games. Young people reported that they mainly engaged in online gaming and social platforms to pass time and to hang out with their friends online. One young woman from Paraisopolis shared that if she did not connect online with her friends, she would not be able to sleep, indicating the importance of digital inclusion for emotional resilience (Furtado, 2021). Whereas, studies found that screentime used for socialization (i.e., texting or video calls) was may be beneficial for mental health and provide a certain degree of stress reduction through strengthened relationships, excessive screen time spent on more “passive” activities such as scrolling, watching TV, or playing video games, may have a negative mental health impact (Doom et al., 2023; Foulkes, 2023; Ho and Lee, 2022).

### 4.2 Case study 2: Indigenous young people in the Brazilian Amazon region

#### 4.2.1 Denialism, misinformation, and the unequal impact of COVID-19 in Brazil

Owing to the lack of adequate and immediate policy-responses at a national level, Brazil's negationist attitude to the COVID-19 pandemic and widespread misinformation under a far-right government led to thousands of avoidable deaths (Hallal, 2021; Paiva et al., 2021; de Camargo and Motta, 2024). This included downplaying the severity of the virus, rejecting scientific evidence, and resisting effective public health measures such as lockdowns and vaccination campaigns—governmental decisions that favored the spread of the virus over its containment (Paiva et al., 2021). In 2020, the mortality rate in Indigenous population was also estimated to be 16% higher than the national rate (Hallal et al., 2020; Horta et al., 2020) and a community-based survey conducted by the Coordination of Indigenous Organizations of the Brazilian Amazon (COIAB) indicated 14% underreporting of COVID-19 cases and 103% underreporting of deaths compared to official government data (Fellows et al., 2021). As a result, communities in situations of vulnerability—Indigenous and non-Indigenous—were often forced to develop their own preventive measures to protect themselves from the virus (Giatti et al., 2021; Olivar et al., 2022).

Young people in Indigenous contexts in the north of Brazil experience key mental health impacts that were exacerbated by the COVID-19 pandemic. There is already intense pressure among Indigenous young people to make the transition to urban life in search for better living conditions with migration often being driven by territorial conflicts (Alves et al., 2023). This often entails rupture from community life, traditional practices, and spiritual connections to the land can also erode protective cultural factors that traditionally support mental wellbeing. Silva et al. (2022) moreover point out that a colonial model persists in various forms of exploitation and domination, hierarchizing and oppressing collectives and individuals based on ethnicity, gender, social class, religion and place of residence. The colonial model manifests in mental health challenges through systemic neglect, the erasure of culturally grounded experiences, and the lack of access to culturally sensitive care. Like young people from São Paulo's favelas, Indigenous young people are therefore confronted with multifaceted stigma, social exclusion, and limited opportunities, often compounded by the erasure of their identities and worldviews within dominant social and institutional systems.

#### 4.2.2 Limitations of the Western mental health model

The survey results from young people in the Brazilian Amazon during the COVID-19 pandemic showed that the idea of “mental health,” as defined by Western cultures, has significant limitations. Western mental health frameworks are inadequate for Indigenous contexts that stress the holistic integration of environmental, community, and spiritual health. In Indigenous contexts, health and wellbeing cannot be dissociated from cultural, environmental, and spiritual contexts (Ullrich, 2019; Ullrich et al., 2022). For the Indigenous communities that took part in the survey, there was no separation between different types of health, as individual health is intricately connected to that of the environment, whether it be elements of nature, community or familiar life. The understanding of mental health must therefore be broadened to include these integrated aspects and other contemporary challenges affecting these young people. Indigenous communities can offer valuable lessons about resilience, adaptability and the importance of a holistic approach to health and wellbeing. In line with the concept of the “pluriverse” we recognize that reality is constituted by many worlds and diverse ways of knowing reality (Querejazu Escobari, 2016). Therefore, any attempts to “translate” the concept of “mental health” across cultures, therefore require a mature understanding of these diverse ways of knowing and a willingness to engage with those many worlds, across human, natural, and spiritual dimensions (Querejazu Escobari, 2016; Núñez, 2019).

#### 4.2.3 Mental health as a “normative concept”

When asked about associations with the term “mental health”, 50% of the young Indigenous people closely related the notion of the “person” and “human being” to the idea of “care”, with a focus on “coexistence” in “community” as important for a broader wellbeing. Indigenous young people overall shared a rather positive notion of “mental health” as a normative concept. In the context of today's world of interconnected crises, there is an urgency to address both the internal (emotional) and external dimensions of the wellbeing and sustainability crisis that young people face for reimagining their futures (Dekeyser, 2022; Wamsler et al., 2021). Among the qualitative responses of Indigenous young people, there were allegations like “*It's very important to talk about this, because we need to keep our psychology in balance”*, and references to mental health in terms of “normal psychological state”, “things related to emotions” and “psychology and consciousness”.

23% of the survey respondents also associated the term with mental illness, using words like “*depression*”, “*sadness*”*, “anxiety*”*, “suicide*”, or “*madness*”. This reflects the emergence of a substantive and escalating youth mental health crisis (McGorry et al., 2025), which can be described as a mental health pandemic alongside the pandemic of COVID-19. Another 14% of the responses associated the notion of mental health with “*illness in the head*”*, “headache*”, “*confusion in the brain*” or “*problems in the head*”.

#### 4.2.4 Intersecting crises: Indigenous youth mental health, violence, and cultural resilience during COVID-19

The origin of mental health problems was pointed out as low self-esteem or “*moments of anxiety*”. Young people narrated different situations where they felt offended and demeaned which impacted their self-esteem. Other also mentioned sexual harassment as a key factor with an influence on mental wellbeing: “*I've been sexually harassed several times, and it disturbs my mind”*. Violence is a historically recurrent and urgent threat to Indigenous ways of living since colonization. In the contemporary Amazon, liquor trade (which is practiced despite being forbidden inside Indigenous lands as homologated by federal law in Brazil), the stigmatization of Indigenous groups, disregard for ancestral identity, sexual abuse, and gender violence (El Kadri et al., 2021) are issues which have been exacerbated by the health emergency of the COVID-19 pandemic. These severity of mental health struggles and the impact on individual and communal life through increased violence are reflected in the following quotes by some of the young respondents:

“*Some young people in the community have mental health problems because of drug use and alcoholism.”*

“*When you feel a rage in your head, you can hang yourself or poison yourself or kill someone else, this happens when you have a mental health problem.”*

For the treatment of mental health problems, in addition to the possibilities of help from health professionals, the component of the cultural context was also present in the search for solutions associated with their ancestral traditions and knowledge, as in the case of the following statements:

“*When I suffer, I go directly to the shaman because in my culture the shamans [pajes] are the doctors that exist in the community.”*

“*I went to the shaman to protect me from all the ills that would affect my personal and emotional life.”*

The transition to a virtual environment during the COVID-19 pandemic indicated that maintaining spiritual and community connections is possible, even in times of physical distancing. However, it is crucial to recognize the limits of these solutions, especially in terms of young people's mental health, where the Western approach often fails to capture the interconnection between individual, community, environmental and spiritual health, which is fundamental for Indigenous populations.

During COVID-19, young people also faced the postponement and cancellation of commemorative events, rituals, dances, generating other stress factors, in addition to financial challenges and health problems. These challenges include the loss of family income, food insecurity and the death of primary caregivers or close family members due to COVID-19 (Doom et al., 2023; Bellier, 2020). Indigenous young people also faced issues of emotional and food insecurity, as well as the threat of death and the concrete reality of the death of family and community members. In this way, aspects and effects of the health crisis on mental health were also highlighted, as in school pressures and the family relationships in the context of the pandemic. Only 17% of the youth reported that they had psychological support provided by the health service, however they identified the need for mental health care especially during the pandemic times, although not all participants sought professional help: “*I've never needed it, but I've been visited because of COVID-19*”, and “*I didn't seek help, but I felt very sad when I lost my grandmother who died because of the coronavirus*”.

#### 4.2.5 Bem viver strategies and Indigenous mental health

**Figure 5:**
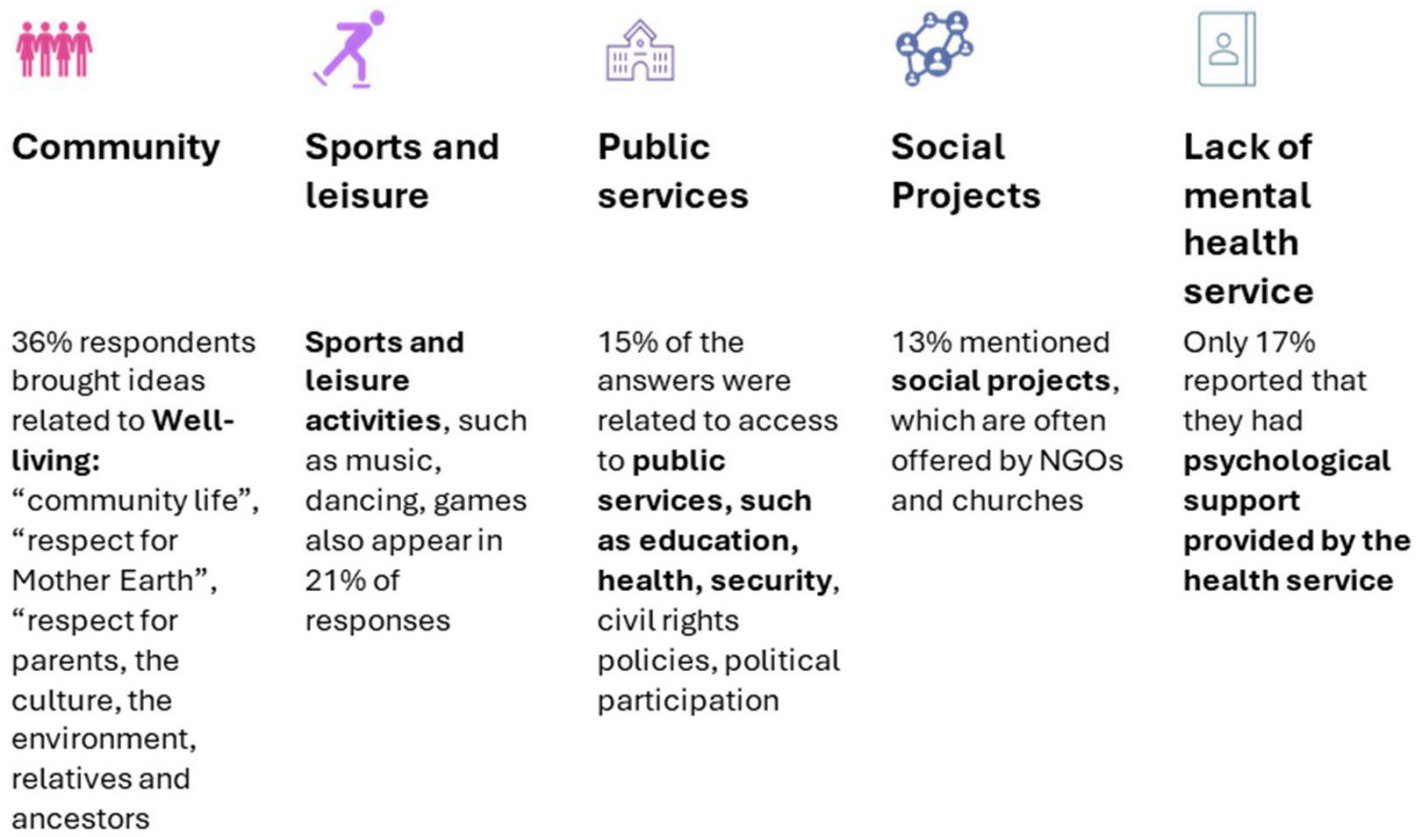
*Bem viver* strategies associated to mental health from Indigenous youth responses.

The FIOCRUZ research also focused on aspects of *bem viver r*elated to the mental health of young Indigenous people, and a summary of these components and strategies is presented in Figure 5.

The challenges of the pandemic in a context of interconnected challenges have revealed that there are possible alternatives for societies to adapt to new and good ways of life when necessary. Among various aspects, the search for connections with their worldviews and cultural context was also emphasized. For example, the proposal to “*create projects to valorise the talents of young Indigenous people so that they can make their people proud*” stands out. The words “community” and “village” were clearly present in terms of living well, and the terms “culture”, “ethnicity”, [Indigenous] “leaders”, “ancestors”, and “mother earth” were also related in this sense. Within the expectation of understanding how to live well in their cultures, there were several references to belonging and a harmonious environment, as in the hope that “*all young people from different ethnicities or cultures respect each other*” or “*if each young person from each village values their culture and ethnicity*” a better coexistence would be possible. Similarly, a holistic view of mental health is also corroborated in statements such as “*wellbeing with oneself and with nature*” and “*cultivating relationships of reciprocity, respect and appreciation for all forms of life*”. The concept of *bem viver* is then essential to respond to these multiple challenges across different dimensions, from urban space to ethnic and identity boundaries, ancestral expectations, and dynamics of power and domination (Verçosa et al., 2023).

Study results show that young Indigenous people live with the most diverse problems that are often no different from those of the non-Indigenous society. Improving the wellbeing of Indigenous young people includes enhancing psychospiritual support, digital inclusion, and the availability of youth leisure spaces. However, in their mental health context, various factors can increase causality, like the subordinate treatment by the state, land conflicts due to agribusiness advances, growing alcohol consumption, child abuse, few prospects for professional or educational inclusion and intergenerational conflicts such as the difficulty of perpetuating traditional ways of life (Souza, 2019). The COVID-19 pandemic has however revealed the resilience and adaptability of Brazil's Indigenous communities, highlighting the importance of collective self-care and *bem viver* strategies (Olivar et al., 2022; El Kadri et al., 2021).

## 5 Discussion

With this paper we have invited a conscious reflection (Figueres and Rivett-Carnac, 2020) on how to be and live well in the face of contemporary polycrises, centring the perspectives of marginalised young people. Young people's diverse, but also collective, experiences have shown that instead of “othering”, we need to make a consistent effort of translation and counter-translation of wellbeing based on local needs and realities. In the words of Nego Obispo (Bispo dos Santos, 2023, translated): “*A river doesn't stop being a river because it flows into another river; on the contrary, it becomes itself and other rivers, it becomes stronger. When we confluence, we don't stop being us, we become us and other people - we yield. Confluence is a force that yields, that increases, that expands*.” (p. 15).

This paper has collated diverse stories and wellbeing strategies of diverse young people, as many of these stories and perspectives have hitherto remained silenced. The importance of talking (more openly) about psycho-spiritual health was expressed by all young people “*to keep our psychology in balance”* (Indigenous youth). The results show that research, policies and interventions to improve youth' emotional resilience must be culturally sensitive and inclusive, recognizing and respecting the traditional wisdom and practices of all communities to promote true good living (Ullrich et al., 2022). As Krenak (2019) encourages, we need to “reforest our imaginations” and embrace a willingness to unlearn old paradigms. The concept of *bem viver* has been helpful in this quest to shape the confluence of lived realities and worldviews for relational understanding on how to live and be well in times of crisis. As researchers (and humans), this requires humility, and a willingness to open to pluriversal ways of rethinking the wellbeing beyond the legacy of Western health models.

### 5.1 Shared experiences: common youth grievances in polycrises

Our findings showed that mental health issues such as anxiety and depression in young people were on the rise already before the COVID-19 crisis (Foulkes, 2023), and especially Indigenous communities suffered from increasing suicide rates. The experiences of Brazilian young people reveal that the COVID-19 pandemic's impact extended beyond physical infection, highlighting the need to address its invisible effects, such as the mental health crisis (Zuccolo et al., 2023; Paiva et al., 2021).

Approaching young people's mental health situations from the analogy of polycrisis (Hoyer et al., 2023) was fundamental in understanding how young people were affected by multiple crises happening simultaneously. Both in Indigenous and urban youth contexts, pre-existing mental health challenges were exacerbated and persisted due compounding and interconnected crises including a lack of leisure spaces, conflictive environments, excessive digital consumption (or digital inclusion), uncertainty about the future, and the (fear of) loss of loved ones. These interconnected and simultaneous crises influence one another through feedback loops that can unpredictably amplify and accelerate their effects. Hence, we argue that supporting and improving youth mental health requires integrated approaches beyond individualistic interventions to strengthen collective and systemic resilience needed to navigate multiple crises (Hoyer et al., 2023; Paiva et al., 2021).

Young people both in the urban periphery of São Paulo and the Amazon region also shared the experience of a period in their life cycle characterized by significant transitions that impacted their emotional and psychospiritual wellbeing. In São Paulo, young people highlighted uncertain futures in times of polycrisis (Börner et al., 2020; Börner, 2023), including uncertainty surrounding their education and the transition to the job market. Similarly, many Indigenous young people in the Amazon faced challenges associated with moving to urban environments, often entailing separation from family and community rituals of spiritual guidance. Consequently, during the pandemic, young people were forced to navigate such sensitive phases of transition to adulthood and increased responsibilities within a context marked by high uncertainty, abandonment, precariousness, and risk (Dekeyser, 2022).

### 5.2 Recognising traditional adaptive strategies

Our findings have shown that beyond a deeper investigation of the systemic factors that contribute to young people's vulnerabilities, it is crucial to explore what strengthens their emotional, spiritual, and psychosocial resilience (Foulkes, 2023; Doom et al., 2023; El Kadri et al., 2021). Although the COVID-19 pandemic has exacerbated social inequalities and injustices, it has simultaneously highlighted young people's ability to engage with and reinvent community and traditional strategies to face threats. In the absence of access to adequate mental health care services in Indigenous communities and urban peripheries, the lived experiences of young people revealed their plurality of adaptation mechanisms to maintain emotional stability. Their experiences stressed the importance of functional networks of care and solidarity within families, communities, and across peer-groups to cultivate resilience and collective hope (Tschakert et al., 2023). These included solidarity and community networks, connection to ancestral traditions, and reinventing pragmatic creativity for engaging with digital leisure, and collective sports and wellbeing activities.

Young people from São Paulo's urban periphery reinforced that in a context where they deemed psychological support “*inaccessible*” because it was expensive and “*everyone else had problems too*” (young girl from Heliopolis), social solidarity networks, physical exercise, or mindfulness practices such as watching the sunset, became important tools for psychospiritual coping and restoration. This was supported by the narrative of one of the participants who used playing soccer as an escape from the mental challenges of fearing for his mother's health who worked in a hospital and had to keep herself isolated from the family. It was through this creative pragmatism that young people found ways to “stay well”. These strategies helped them transcend the context of crisis that they found themselves in, while finding ways to cope with the lack of professional mental health support and limited possibilities of restorative human-nature connection (Sahni and Kumar, 2021).

### 5.3 Policy recommendations

At a practical and policy level, young people's lived experiences show the importance of integrating young people's psycho-spiritual wellbeing as a cross-cutting issue across all education, health, and urban planning policies. Both in the urban periphery of São Paulo and in the Brazilian Amazon region there is an urgency to redirect more resources to mental health support interventions directed at young people in situations of vulnerability, prioritizing their access to education, leisure, and youth wellbeing services. At the same time, interventions and policies need to be bottom-up and grounded in local realities and cultural needs. We therefore call for:

- Integrating diverse epistemologies for youth-led emotional and psycho-spiritual wellbeing as a **cross-cutting priority** in mental health services, interventions, and educational programmes.
- Developing **localized, community-driven wellbeing hubs** in vulnerable areas, co-designed with young people and grounded in their cultural and ecological realities and diverse knowledge systems, based on the principles and practices of ***bem viver***.
- Introducing participatory frameworks for **emotion-centered education** that promote youth agency and cultivate self-awareness, compassion, behavioral regulation, emotional resilience, and critical hope (such as the EMPOWER-framework developed by Börner, 2023).

This includes supporting young people to acknowledge, validate, self-regulate, and actively engage with their emotions, rather than numbing and distracting (Börner, 2023; Tschakert et al., 2023; Wamsler et al., 2021) through maladaptive coping mechanisms such as substances, gaming, or social media.

Psychosocial and spiritual support is needed not only during acute but also chronic collective crises, to help young people process grief, share their loss, and sustain a sense of meaning and purpose (Börner, 2023; Coppola et al., 2021; Jaeger et al., 2021; Tschakert et al., 2023). Therefore, we suggest that mental health education and interventions that improve young people's “inner” states of wellbeing through practices of “self-reflection and awareness, behavioral regulation, [and] compassion” (Wamsler et al., 2021: 6) need to go hand in hand with community-oriented strategies, to improve self-efficacy and openness to new perspectives and experiences (Tschakert et al., 2023). This also includes alleviating wider stigma—i.e., growing up in the deprived territories during formative years of identity-formation—while recognizing the plurality of lived experiences of marginalised young people, including age, gender, ethnic, and cultural diversity.

Youth wellbeing can be reimagined through diverse forms of knowledge and by challenging internalized beliefs and narratives surrounding the diagnosis and prevention of mental health issues (El Kadri et al., 2021). Dealing with psychosocial and mental health challenges requires surpassing individualistic approaches outside social and cultural contexts (Paiva et al., 2021). Therefore, we suggest integrating *bem viver* principles into the development of mental health services to acknowledge the importance of traditional cosmovisions and relational practices that stress interconnectedness of the wellbeing of individuals with the wellbeing of communities and the natural world (Kantner and Peixoto, 2023). Particularly for Indigenous young people, mental health interventions for prevention and treatment need to be community-based rather than prioritizing individual solutions, based on the resources available in the environment and the community (Ullrich, 2019; Ullrich et al., 2022). Integrating the principles of *bem viver* into public health services, mental health education, and interventions, can be a powerful tool for reorienting the way mental health services are organized in Brazil, which are currently excessively centered on health units and often very distant from localized lived realities.

In today's context of multiple interconnected crises, *bem viver* can help build more conscious wellbeing frameworks through relational paradigms that reclaim the interconnectedness of wellbeing and the kinship between individuals, society and nature (Figueres and Rivett-Carnac, 2020; Hodgetts, 2023). Such adaptive and counter-hegemonic perspective of effective interventions and policies, grounded in everyday cultural and social realities and open to the diversity of knowledges can also foster greater resilience in the face of contemporary uncertainties and challenges (Bronk and Jacoby, 2016; Davies and Hobson, 2023).

### 5.4 Directing future youth wellbeing scholarship: a confluence of knowledges

The exercise of reflecting on the lived experience of both Indigenous and non-Indigenous Brazilian young people in situations of historical exclusion and vulnerability shows that recognizing the confluence diverse realities goes beyond a “comparison” or creation of knowledge hierarchies (Querejazu Escobari, 2016; Bispo dos Santos, 2023). Rather, what is needed are more bridges between diverse knowledges and lived experiences by listening, learning and acting with each other (Askins, 2018). When we no longer dismiss or minimize each other's experiences (Börner, 2023), young people's collective experiences can remind us of the importance of community life, activities such as sports, dance, and music, education, and participation as central to their emotional wellbeing, along with the wellbeing of loved ones.

This paper has made a first attempt to create such a space for reflection, (un-)learning and co-creation. Bringing together public health researchers, psychologists, and geographers from the Global South and North, along with Indigenous and non-Indigenous youth knowledge, has initiated a confluence of knowledges. The collaboration has encouraged questioning and rethinking how we understand and address young people's wellbeing and what it means to live a good life in the context of polycrises. Weaving together global and local dimensions of wellbeing and well-living, based on diverse methodologies, ontologies and lived experiences, we have attempted to celebrate diversity alongside the collective humanity, based on relational paradigms of care, mutual respect and learning.

Limitations of the study however include the diverse methodologies for data collection which, while able to bring a confluence of knowledges around young people's wellbeing, limited exploring shared experiences through immediate youth dialogue between Indigenous and urban young people. For future youth wellbeing scholarship, we suggest enabling joint reflections on both shared and different lived experiences rather than perceiving them as “competing or exclusionary perspectives” (Börner, 2024, p. 45). We therefore call for co-producing shared knowledge on healing and wellbeing with *both* Indigenous and non-Indigenous young people that cultivate collective wisdom, curiosity, and compassion, through the willingness to learn from and with each other (Börner, 2023; Ullrich et al., 2022). Only then can societies fully realize *bem viver*—a way of living well together in community and the collective—grounded in cultural identity, spiritual guidance, belonging, reciprocity, and respect for the plurality of worldviews (El Kadri et al., 2021).

## 6 Conclusions

We have argued that youth wellbeing in Brazil is shaped by intersecting and compounding crises—including systemic inequalities, environmental degradation, cultural loss, and the increasing precarity of education and employment. In the context of COVID-19, these conditions have profoundly affected how young people understand their lives, imagine their futures, and respond to adversity. Our study has made a significant contribution to questioning and reshaping understandings of youth wellbeing from a decolonial perspective to reflect (on) the pluriversal structural, cultural, and relational dimensions of youth wellbeing. Grounded in youth-led research conducted with both urban and Indigenous young people from São Paulo and the Brazilian Amazon during and after the pandemic, this confluence of diverse youth voices enabled challenging dominant, individualistic Western mental health models. Rooted in the relational philosophy of *bem viver* (good living), our research identified important wellbeing practices that emphasized collective care, reciprocity, emotional interdependence, and connection to their communities. While the pandemic intensified young people's psychosocial suffering—including anxiety, grief, and depression—young people responded creatively through self-organized support networks, creative pragmatism, and re-engagement with ancestral and community-based knowledge. Through the lens of *bem viver* our study has highlighted that youth wellbeing must be understood through youth-led, relational frameworks that prioritize their lived experience and cultural plurality. This reorientation calls for future research, education, policies, and interventions to be co-created with diverse young people—both Indigenous and non-Indigenous—embracing *bem viver* as a foundation for healing, belonging, and imagining more just and collective futures.

## Data Availability

The raw data supporting the conclusions of this article will be made available by the authors, without undue reservation.
